# Molecular modeling analysis for functionalized graphene/sodium alginate composite

**DOI:** 10.1038/s41598-024-64698-x

**Published:** 2024-06-27

**Authors:** Hanan Elhaes, Asmaa Ibrahim, Osama Osman, Medhat A. Ibrahim

**Affiliations:** 1 Physics Department, Faculty of Women for Arts, Science and Education, Ain Shams University, Cairo, 11757 Egypt; 2 Spectroscopy Department, National Research Centre, 33 El-Bohouth St., Dokki, Giza 12622 Egypt; 3 Molecular Modeling and Spectroscopy Laboratory, Centre of Excellence for Advanced Science, National Research Centre, 33 El-Bohouth St., Dokki, Giza 12622 Egypt

**Keywords:** Graphene, Sodium alginate, DFT, B3LYP, Electronic properties, Biophysics, Materials science

## Abstract

This study examined the functionalization of graphene with easily ionizable elements, such as lithium, and subsequently its interaction with the biopolymer sodium alginate (SA), to highlight its potential for biomedical applications. Utilizing Density Functional Theory (DFT), the research comprehensively investigated the structural, electronic, and spectroscopic properties of these graphene-based composites. The electronic properties of functionalized graphene were investigated using DFT at the B3LYP/6-31G(d,p) level. Among the various configurations studied, graphene exhibited weak interaction with two lithium atoms, displaying the highest reactivity in terms of total dipole moment (TDM) at 5.967 Debye and a HOMO/LUMO energy gap (ΔE) of 0.748 eV. Electrostatic potential mapping revealed that graphene when enhanced with lithium and three units of SA, exhibited an augmented potential density on its surface, a finding corroborated by other investigated physical properties. Notably, the configuration of graphene/3SA/Li, with weak interaction occurring at two side carbons, demonstrated the highest reactivity with a TDM of 15.509 Debye and ΔE of 0.280 eV. Additionally, a shift in the spectral characteristics of graphene towards lower wavenumbers was observed as lithium and SA interacted with the graphene substrate. The PDOS plot for Graphene/3SA/Li, showed the highest contribution in the HOMO orbitals was equally from lithium, sodium, hydrogen, and oxygen, while the lowest contribution was from carbon. This computational analysis provides comprehensive insights into the functionalized graphene systems, aiding in their further development and optimization for practical biomedical use.

## Introduction

Emerging from recent advancements, two-dimensional (2D) nanomaterials exhibit exceptional promise for biomedical applications compared to traditional nanoparticles. This is attributed to their unique physicochemical properties, including high surface area, exceptional physiological stability, favorable biocompatibility, facile modification capabilities, and inherent multifunctional potential^1^. Within this exciting class of advanced nanomaterials, graphene, a groundbreaking 2D carbon material, has garnered significant research interest in recent years^2,3^. The single-atom-thick layer of carbon atoms covalently bonded in a hexagonal lattice, known as graphene (G), is the foundational member of the 2D nanomaterial family. This sp2-hybridized structure grants graphene an exceptional surface area and unique electrical and thermal properties, making it highly versatile for various applications^4,5^. Graphene boasts a remarkably high surface area, allowing for excellent interaction with adsorbed molecules. Additionally, it demonstrates high carrier mobility for both electrons and holes^6^. Incorporating graphene into a composite material significantly improves its surface properties, particularly when the application relies heavily on surface performance^7^. Strategic incorporation of various atoms into the graphene structure, a process known as doping, can be employed to optimize its properties for specific applications. For instance, nitrogen-doped graphene exhibits promising potential for biosensor development, as mentioned previously^8^. Building on the earlier point about nitrogen doping, it is worth noting that this process can also significantly improve graphene’s electronic properties, making it a valuable material for supercapacitor applications^9^. Due to their exceptional surface area and unique electronic properties, both advanced graphene and silicon have demonstrated the potential for gas sensing applications^10^.

Molecular modeling, a powerful computational technique, allows scientists to investigate the physical and chemical properties of various systems and molecules. In this context, Density Functional Theory (DFT) was employed to explore potential methods for tailoring the characteristics of graphene quantum dots (GQDs)^11^. Results revealed that chemical functionalization with specific groups could be a powerful method for manipulating the properties of GQDs. Notably, the study found that attaching carboxyl groups to the edges of GQDs significantly enhanced their electronic properties^12^. Other important applications of graphene were indicated by DFT study^13,14^. GQDs are emerging as promising candidates for removing heavy metals from water as a result of their unique combination of surface and electronic properties. A computational approach based on DFT predicts that GQDs can be tailored (functionalized) to target and eliminate harmful organic pollutants from aquatic environments^15^. Studies have demonstrated the effectiveness of functionalized GQDs in removing atrazine from contaminated water. Researchers investigated another approach by incorporating WO_3_ and PVDF onto graphene oxide^16^. Computational calculations, including TDM and molecular electrostatic potential, suggest a potential interaction between the GO/WO_3_/PVDF nanocomposite and glucose molecules. These findings support the application of this nanocomposite as a biosensor. Furthermore, Berisha et al.^17^ demonstrated the effectiveness of DFT in studying structural changes in similar molecules, highlighting its potential as a tool to monitor modifications within the nanocomposite itself. Scientists investigated the potential interactions that might occur between aryl diazonium cations and graphene oxide^17^. A study explored that the calculated interaction energy was up to − 45 kcal/mol, which indicated the potential for bonding or reactivity between aryl diazonium cations and graphene oxide surfaces.

DFT calculations were employed to analyze the impact of doping graphene and silicon nanosheets on various elements (Li, Be, B, N, O, and F) on their electrical, thermodynamic, and optical properties^18^. The element lithium (Li), renowned for its remarkable lightness, is the only metal buoyant enough to float on water. Furthermore, it boasts exceptional electrochemical activity and a remarkably high specific heat capacity^19^. These make Li required by many industries in the present century^20^. Li finds applications across a diverse range of industries, including ceramics^21^, glass^22^, chemicals^23^, refrigerants^24^, and pharmaceuticals^25^. The most prominent application, however, remains in the field of batteries^26,27^. Li maintains its status as the preferred medication for preventing bipolar disorder. It effectively hinders the return of both manic and depressive episodes, offering long-term stability^28^. In addition to its established effectiveness, long-term Li treatment can offer a range of potential benefits, including reducing suicidal tendencies, fighting viruses, and even protecting against dementia^29^. These unique characteristics solidify Li’s position as one of the most remarkable elements for material functionalization. Its exceptional ability to lose an electron (ionization) makes it ideal for achieving superior energy and power densities in high-demand battery applications, such as electric vehicles and backup power systems^30^.

Enhancement of graphene is an important step for better functionalization; this enhancement could be achieved with both theoretical and experimental efforts. Pristine is introduced in many studies for graphene. A pristine single-walled boron nitride nanotube could be enhanced with both Al and Ga for possible detection of the 1-chloro-1,2,2,2-tetrafluoroethane gas molecule. DFT-obtained energy calculations and population analyses show that by adsorbing the gas molecules on the surface of modified pristine, they are interacting as strong chemical adsorption^31^. DFT was used to examine a possible interaction between serine (C_3_H_7_NO_3_) and fullerene nanocages, C_60_, in vacuum. The overall aim is to reveal the nature of the intermolecular interactions. Results indicated that the ΔEs were C_60_: 5.996 eV, C_59_Si: 5.309 eV and C59Ge: 5.188 eV at DFT:B3LYP-D3/6–311 G(d) level. Furthermore, the adsorption increased when serine amino acid was interacted with doped C_60_, which paves the way towards the possible design and implementation of nanocarrier for detecting amino acid^32^. DFT supported experimental findings for the synthesis of a bisquinoline A-D-A with high planarity, aggregation-induced emission, and interesting photophysical and nonlinear properties by Knoevenagel condensation^33^. The HOMO/LUMO energies show values of − 5.39 eV and − 3.67 eV, respectively, and the electrochemical band gap is − 1.72 eV. These values indicate that bisquinoline is an attractive organic semiconductor whose energy levels could match those required for photovoltaic applications. The incorporation of functionalized and pristine nanostructures for cancer therapy offers substantial prospects to curb the persistent problems of ineffective drug administration and delivery to target sites. The functionalized pristine was used as an efficient nanocarrier for 5-fluorouracil (5FU) and studied with B3LYP-GD3(BJ)/6–311 +  + G(d,p) model^34^. The calculated electronic properties effectively account for all adsorption interactions of the drug on the investigated surfaces. Pristine C_24_ was utilized as a model molecule for graphene, then further encapsulated with Mg, K, Ca and doped with Ni to enhance the surface for sensing some gases such as CO_2_, NO_2_ and SO_2_ respectively^35^. The potential of heteroatoms (B, N, S)-doped quantum dots as enhanced nanocarriers or delivery surfaces for isoniazid is studied using DFT. Doped quantum dot surface was enhanced to test its ability for sensitivity and delivery properties of isoniazid^36^. Quantum dots are further doped with As and Co to enhance the surface for better sensing performance. DFT was used with three functionals, and the obtained results indicated that the calculated adsorption energies show thermodynamic favorability and spontaneity^37^. Enhancing the surface of graphene oxide (GO) is done to produce biomarkers. So that, a film was papered by GO enhanced with ZnO and then embedded in poly(3,4-ethylenedioxythiophene) (PEDOT). GO/Zn/PEDOT was able to detect Acyl homoserine lactones (AHLs) with adsorption enthalpies of − 4.02, − 4.87 and − 4.97 kJ/mol, respectively^38^. DFT is conducted to examine the possible application of the carbon nanocone (CNC) layer with topological defects as an anode material in the performance of Li-ion batteries (LIBs). Results indicated that the Li atom exhibits fast diffusion on the surface of both the Stone–Wales (SW)-defect-filled CNC and pristine CNC layers with energy barriers of 0.38 and 0.32 eV, respectively. It could be concluded that, the CNC shows promising applications as an anode in LIBs^39^.

On the other hand, sodium alginate (SA) is a natural, non-toxic anionic polysaccharide, meaning it is a sugar molecule with a negative charge derived from living organisms. This biocompatible biopolymer boasts high water absorption capabilities and forms non-covalent bonds, which strengthens its ability to create hydrogels. These hydrogels hold significant promise for various biomedical applications^40^. Beyond its hydrogel capabilities, SA also demonstrates remarkable potential as a film-forming material^41^. Besides its wide range of applications, it is also described as an easy-to-handle polymer and an eco-friendly^42^. Moreover, enhancing the electronic properties of SA can improve its ability to encapsulate drugs, facilitating controlled and targeted drug delivery. By modifying its electronic properties, researchers can accurately determine the release kinetics of drugs from alginate-based carriers, ensuring optimal therapeutic efficacy and minimizing side effects^43,44^. Alginate-based dressings are commonly used for wound management due to their moisture-retention properties and ability to promote wound healing. Enhancing the electronic properties of SA can improve its antimicrobial activity and accelerate the healing process by providing a conducive environment for tissue regeneration while preventing infections^45,46^.

Building on these findings, the current study proposes a method to enhance both the functionality and biocompatibility of graphene. This approach involves first functionalizing graphene with a readily ionizable element like Li. Afterward, the biocompatible biopolymer SA, is introduced to further improve biocompatibility. To explore this concept, the study employed DFT at the B3LYP level to investigate the properties of graphene. The process involved sequential functionalization: graphene, then graphene-Li (various interaction schemes), and finally, the graphene-Li-SA composite. Key properties like TDM, ΔE, molecular electrostatic potential, and Infrared spectra were all calculated for these model molecules. Finally, density and projected density of states (DOS and PDOS) were plotted for better understanding of electronic properties.

## Calculations details

All calculations were conducted using Gaussian 09 (G09)^47^ software. This software is installed on a personal computer at the Molecular Modeling and Spectroscopy Laboratory at the National Research Centre's Centre of Excellence for Advanced Sciences. The first step involved optimizing the model molecules using DFT using a hybrid exchange–correlation functional B3LYP^48–50^. A split valence basis set 6-31G(d,p) was used to form B3LYP/6-31G(d,p) model for the present calculations. Following optimization of each structure, several key properties were analyzed: TDM, ΔE, and molecular electrostatic potential. Both Infrared (IR) and Raman spectra were calculated for the optimized structures, at the same level of theory. The DOS and PDOS were also studied at the same level of theory.

## Results and discussion

### Building model molecules

**Figure 1:**
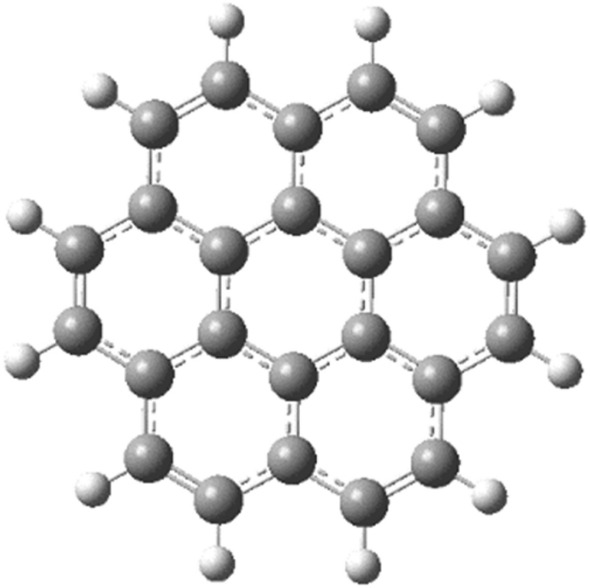
Model molecule for graphene calculated at DFT:B3LYP/6-31g(d,p) level of theory.

**Figure 2:**
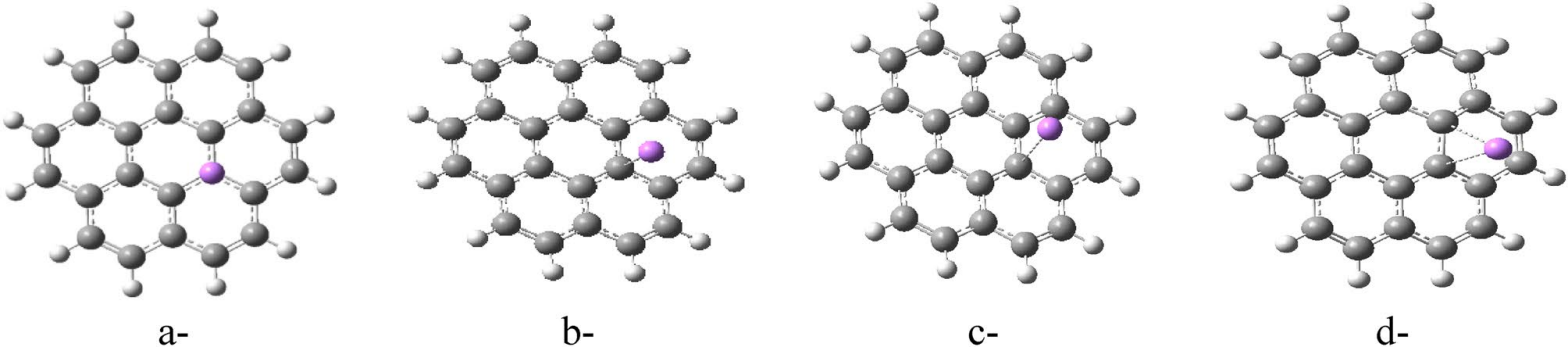
Model molecules of graphene interacted with Li forming G/Li in different cases (**a**) one carbon atom is substituted with Li atom, (**b**) one Li atom is complexed with carbon atom, (**c**) one Li atom is weakly interacted with carbon atom, (**d**) one Li atom is weakly interacted with two carbon atoms. All structures are calculated at DFT:B3LYP/6-31g(d,p) level of theory.

**Figure 3:**
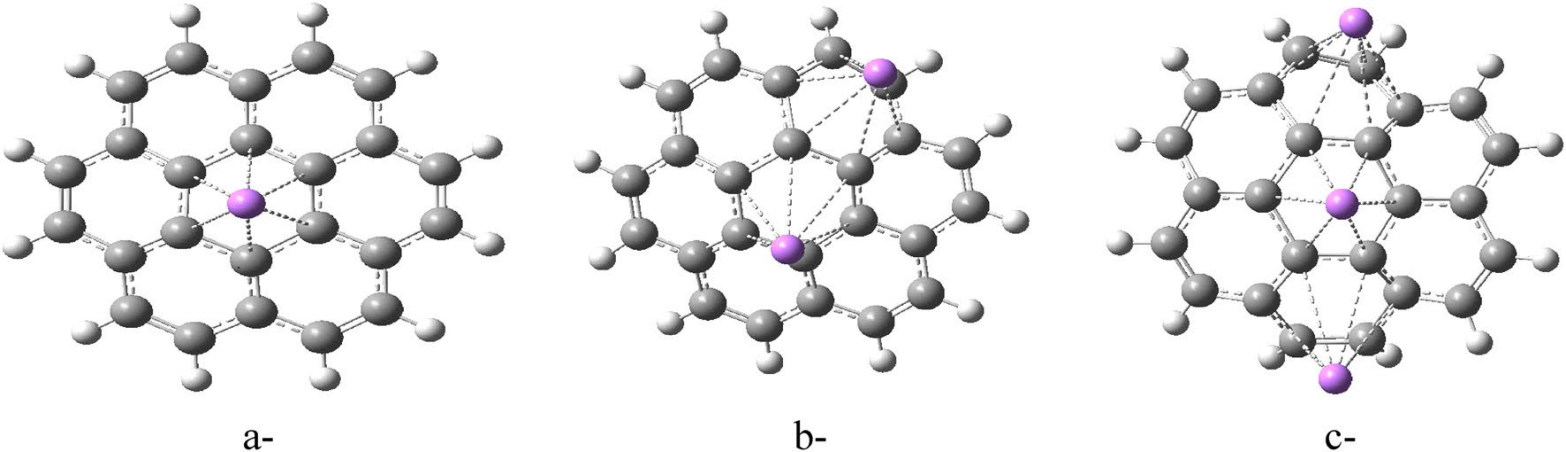
Model molecules of graphene interacted with Li forming G/Li in different cases (**a**) one Li atom is weakly interacted with five carbon atoms, (**b**) two Li atoms each one is weakly interacted with five carbon atoms, (**c**) three Li atoms each one is weakly interacted with five carbon atoms. All structures are calculated at DFT:B3LYP/6-31g(d,p) level of theory.

**Figure 4:**
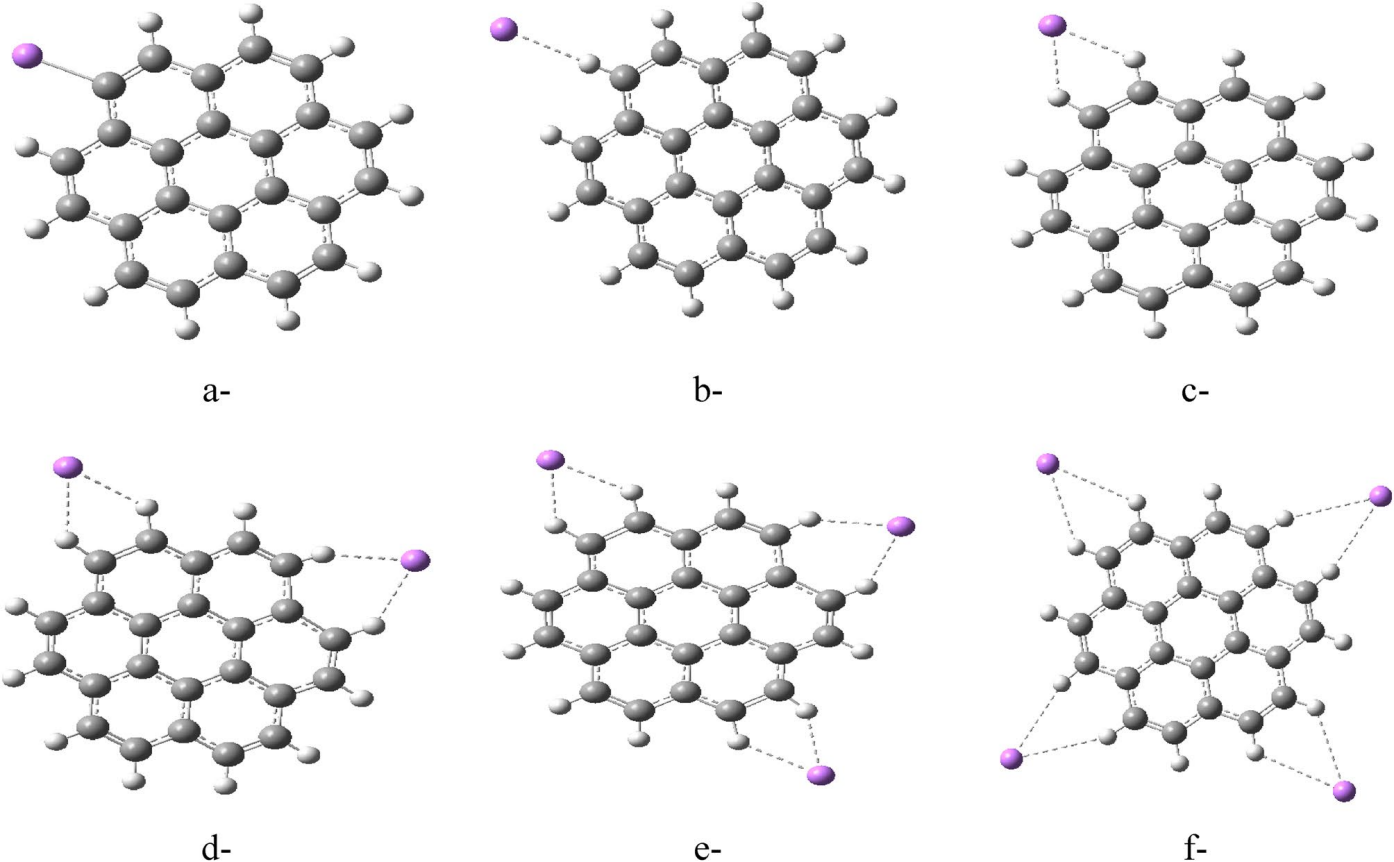
Model molecules of graphene interacted with Li forming G/Li in different cases (**a**) one Li atom is complexed with side carbon atom, (**b**) one Li atom is weakly interacted with one side carbon atoms, (**c**) one Li atom is weakly interacted with two side carbon atoms, d- two Li atoms each one is weakly interacted with two side carbon atoms, (**e**) three Li atoms each one is weakly interacted with two side carbon atoms and (**f**) four Li atoms each one is weakly interacted with two side carbon atoms. All structures are calculated at DFT:B3LYP/6-31g(d,p) level of theory.

The study employed a model molecule containing 24 carbon atoms to represent graphene (Fig. 1). They then explored various ways that Li could interact with this model of graphene. These interaction schemes are presented in Figs. 2, 3, and 4. Figure 2 depicts scenarios where Li directly replaces a carbon atom in graphene (2a), forms a complex with a single carbon (2b), or interacts weakly with one (2c) or two carbon atoms (2d). Figure 3 explores additional interactions: a single Li atom weakly bonded to five carbon atoms (3a), two Li atoms each interacting with five carbons (3b), and three lithium atoms weakly bonded to five carbons each (3c). Finally, Fig. 4 illustrates the possibility of Li interacting along the edge of the graphene molecule. Continuing the exploration of Li interaction with graphene (presented in Figs. 2 and 3), Fig. 4 focuses on scenarios where Li interacts along the edges of the molecule. Figure 4a depicts a single Li atom forming a complex with a side carbon atom. Figures 4b through 4f illustrate various weak interactions: a single Li atom with one side carbon (4b), a single Li atom with two side carbons (4c), two Li atoms each with two side carbons (4d), three Li atoms each with two side carbons (4e), and finally, four Li atoms each with two side carbons (4f).

### Studying physical parameters of graphene/Li

Table 1 summarizes the calculated TDM and ΔE for the studied graphene models using a computational method called DFT: B3LYP/6-31g(d,p). These values are important indicators of a molecule’s reactivity. Lower ΔE and higher TDMs generally suggest higher reactivity. The table shows that pristine graphene (without Li) has a TDM of 0.000 Debye and a ΔE of 4.035 eV. When Li is introduced and interacts with graphene in various ways (13 different schemes were investigated), both TDM and ΔE tend to change. This indicates that Li generally increases the reactivity of graphene. According to the table, the most significant increase in reactivity is observed for the structures where Li weakly interacts with either one or two carbon atoms in the middle of the graphene sheet (G/Li and G/2Li structures). These structures exhibit the highest TDM (5.567 Debye and 5.967 Debye, respectively) and the lowest ΔE (1.070 eV and 0.748 eV, respectively). This suggests that these specific interaction schemes between Li and graphene lead to the greatest enhancement in reactivity.

**Table 1:** TDM as Debye, ΔE as eV for the studied structures which calculated at DFT:B3LYP/6-31g(d,p).

Structure	TDM	ΔE
G	0.000	4.035
G/Li “substituted with one carbon”	1.427	1.172
G/Li “complexed with one carbon”	1.115	0.954
G/Li “weakly with one carbon”	5.060	1.272
G/Li “weakly interacted with two carbons ”	5.567	1.070
G/Li “weakly interacted with five carbons”	4.151	1.148
G/2Li “weakly interacted with five carbons”	0.824	1.311
G/3Li “weakly interacted with five carbons”	0.770	0.918
G/Li “complexed with side carbon”	7.586	3.314
G/Li “weakly interacted with side carbon”	0.184	1.265
G/Li “weakly interacted with two side carbons”	0.162	2.181
G/2Li “weakly interacted with two side carbons”	5.967	0.748
G/3Li “weakly interacted with two side carbons”	0.206	0.168
G/4Li “weakly interacted with two side carbons”	0.021	0.654

**Figure 5:**
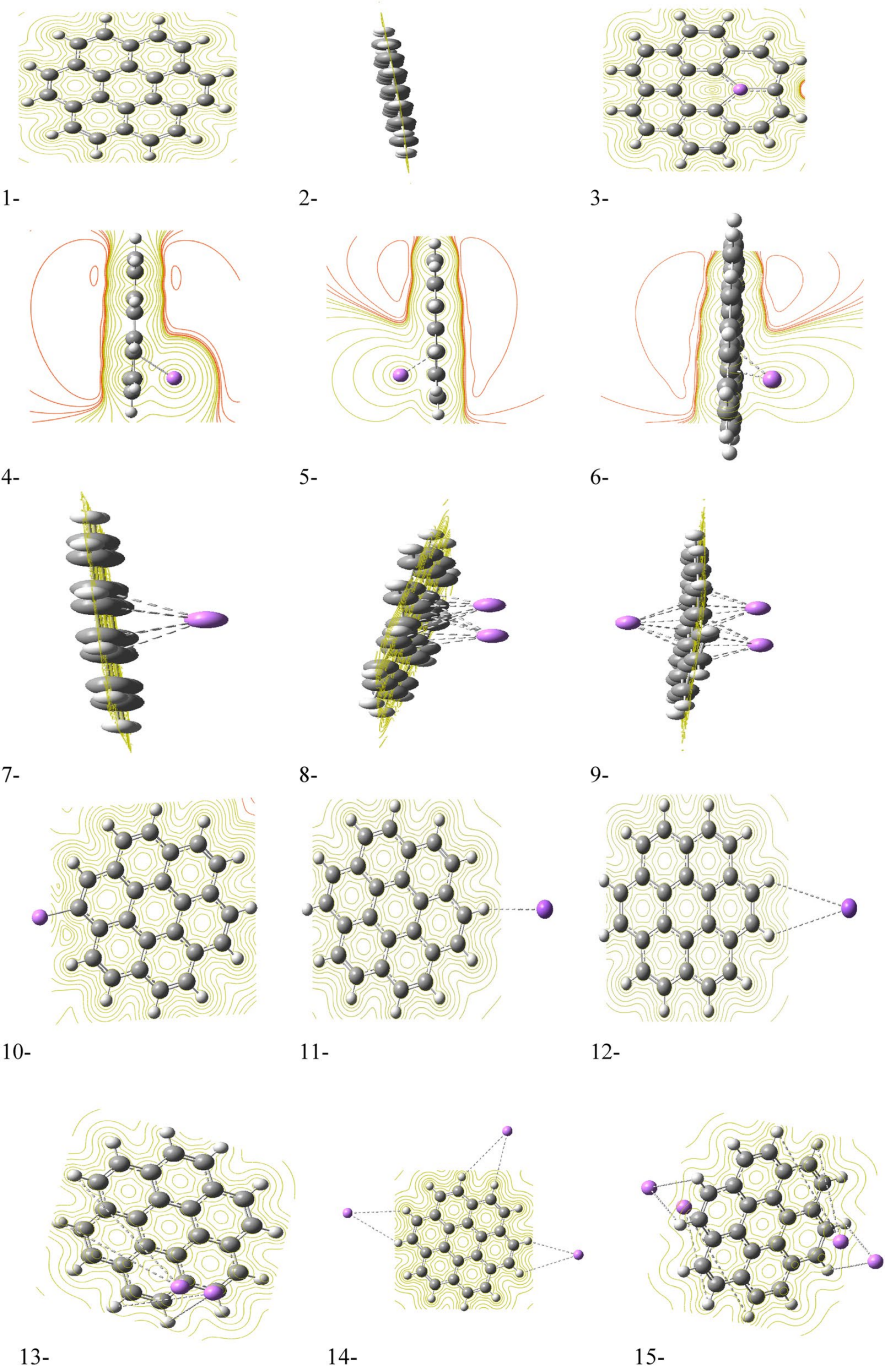
Molecular electrostatic potential maps for the studied model molecules of 1- Graphene; 2- Graphene side view, 3- one carbon atom is substituted with Li atom, 4- one Li atom is complexed with carbon atom, 5- one Li atom is weakly interacted with carbon atom, 6- one Li atom is weakly interacted with two carbon atoms. 7- one Li atom is weakly interacted with five carbon atoms, 8- two Li atoms each one is weakly interacted with five carbon atoms, 9- three Li atoms each one is weakly interacted with five carbon atoms. 10- one Li atom is complexed with side carbon atom, 11- one Li atom is weakly interacted with one side carbon atoms, 12- one Li atom is weakly interacted with two side carbon atoms, 13- two Li atoms each one is weakly interacted with two side carbon atoms, 14- three Li atoms each one is weakly interacted with two side carbon atoms and 15- four Li atoms each one is weakly interacted with two side carbon atoms. All structures are calculated at DFT:B3LYP/6-31g(d,p) level of theory.

**Figure 6:**
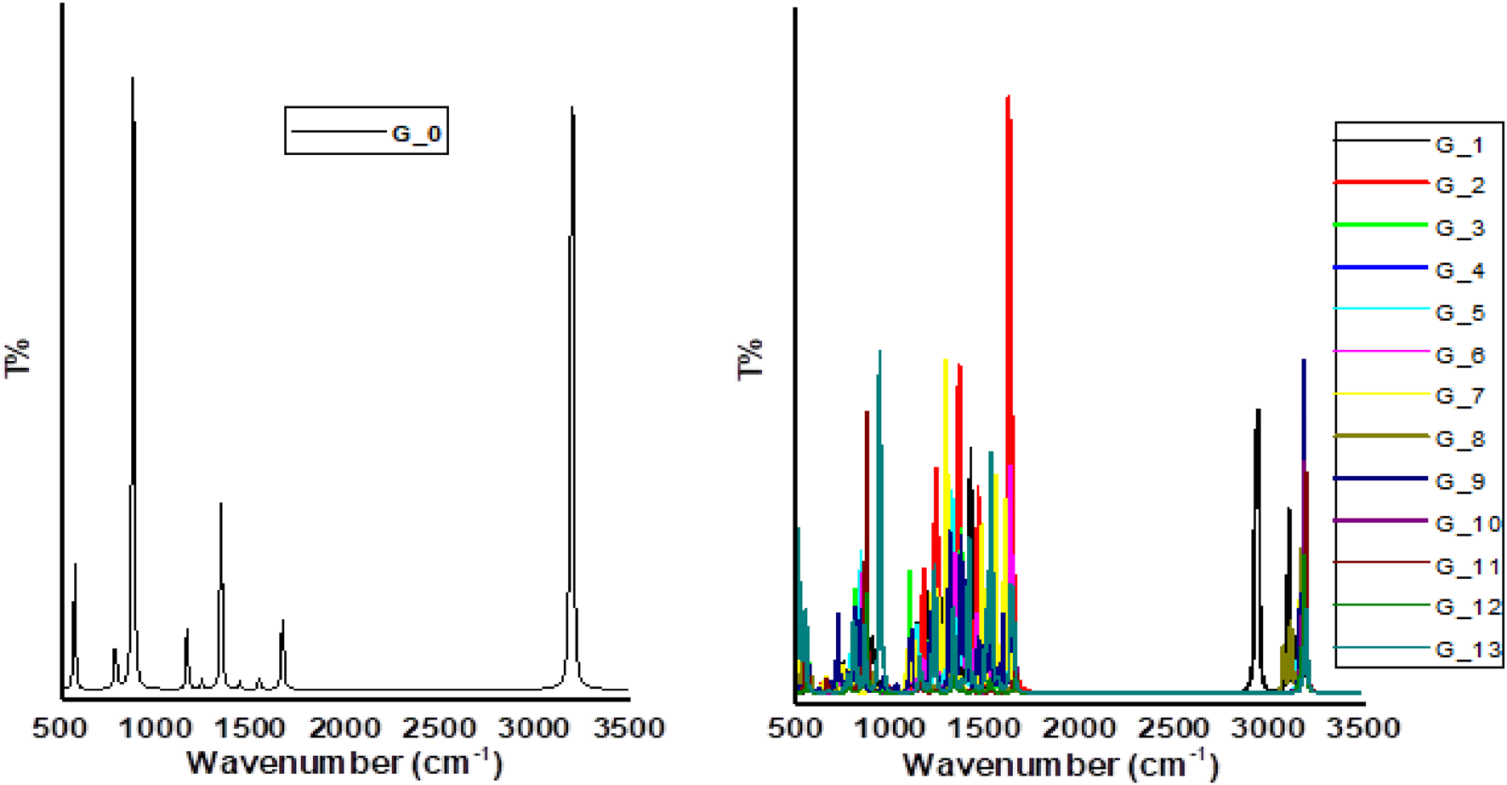
IR spectrum for the studied graphene as well as graphene/Li composites. 1- Graphene (G_0); 2- one carbon atom is substituted with Li atom (G-1), 3- one Li atom is complexed with carbon atom (G-2), 4- one Li atom is weakly interacted with carbon atom (G-3), 5- one Li atom is weakly interacted with two carbon atoms (G-4). 6- one Li atom is weakly interacted with five carbon atoms (G-5), 7- two Li atoms each one is weakly interacted with five carbon atoms (G-6), 8- three Li atoms each one is weakly interacted with five carbon atoms (G-7). 9- one Li atom is complexed with side carbon atom (G-8), 10- one Li atom is weakly interacted with one side carbon atoms (G-9), 11- one Li atom is weakly interacted with two side carbon atoms (G-10), 12- two Li atoms each one is weakly interacted with two side carbon atoms (G-11), 13- three Li atoms each one is weakly interacted with two side carbon atoms (G-12) and 14- four Li atoms each one is weakly interacted with two side carbon atoms (G-13). All structures are calculated at DFT:B3LYP/6-31g(d,p) level of theory.

**Figure 7:**
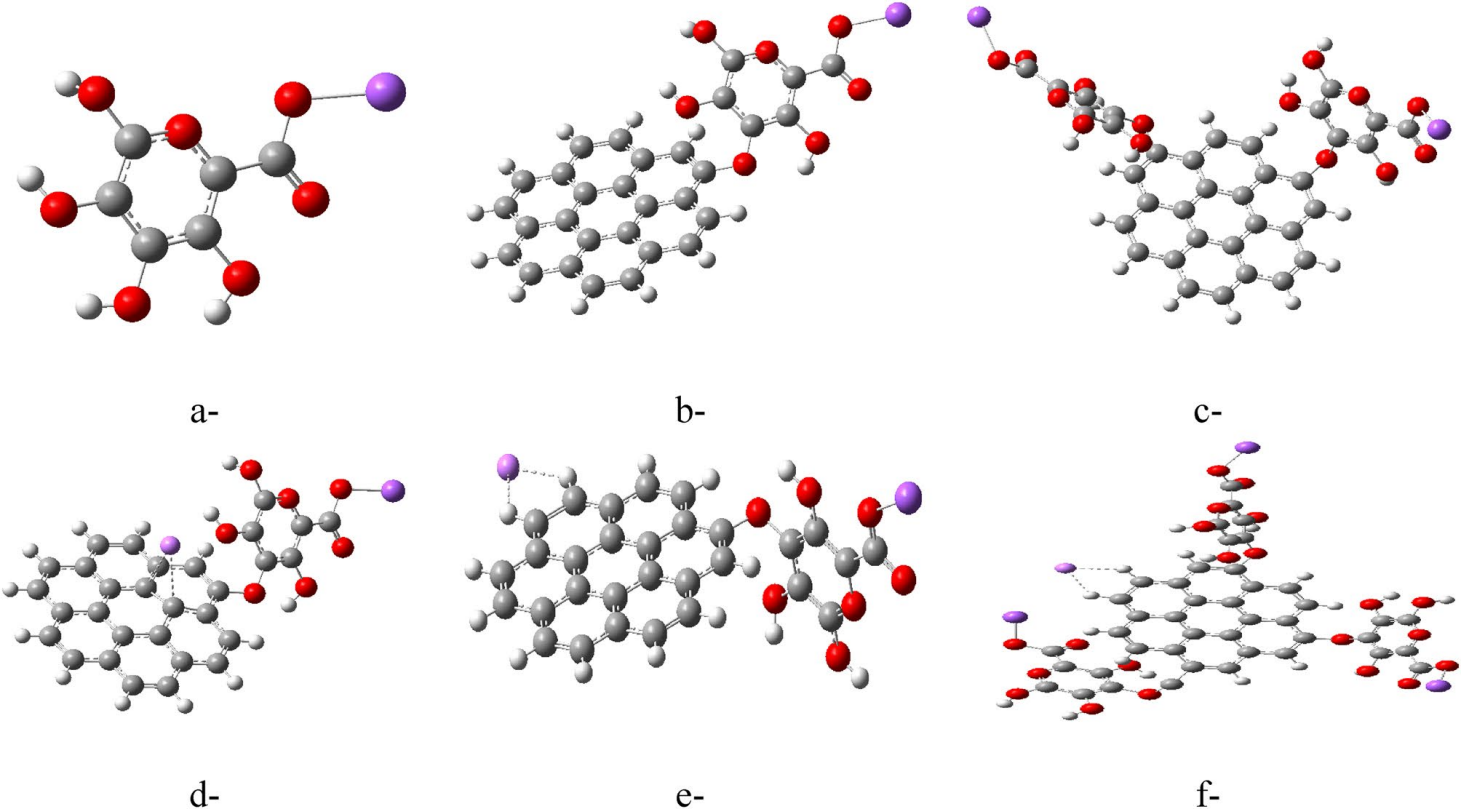
Model molecules for (**a**) Sodium alginate (SA), (**b**) Graphene/SA, (**c**) Graphene/2SA, (**d**) Graphene/SA/Li weakly interacted with two inner carbons, (**e**) Graphene/SA/Li weakly interacted with two side carbons and (**f**) Graphene/SA/Li weakly interacted with two side carbons. All structures are calculated at DFT:B3LYP/6-31g(d,p) level of theory.

Another valuable tool for understanding a molecule's reactivity is the molecular electrostatic potential (MESP) map. This map uses a color scheme to represent the potential energy of electrons at different regions of the molecule. Red indicates areas rich in electrons (negative potential), yellow represents neutral areas (zero potential), and blue indicates areas that tend to accept electrons (positive potential). MESP maps can be used to predict how a molecule might interact with its surrounding environment. Figure 5 presents the MESP maps calculated for the studied model molecules using the DFT: B3LYP/6-31g(d,p) method. Figure 5-1 depicts the MESP map of pristine graphene. As expected, the entire surface appears yellow, indicating a neutral electrostatic potential (zero charge). This is further confirmed by the side view of graphene shown in Fig. 5-2. However, when Li is introduced and interacts with graphene (Fig. 5-3), the MESP map changes significantly. In this specific case, where a single lithium atom forms a complex with a carbon atom, the area near the lithium atom appears red. This indicates a more negative potential in this region due to the presence of electron-rich Li. Continuing the analysis of MESP maps (Fig. 5), Fig. 5-4–6 depict scenarios where Li weakly interacts with graphene. In all three cases, the surface near the Li atom exhibits a red color, indicating a negative electrostatic potential due to the presence of electron-rich Li. This is consistent with the increased reactivity observed for these structures based on the data in Table 1. As shown in the table, these three interaction schemes (one Li interacting with one, two, or five carbon atoms) possess the highest calculated TDM of 5.060 Debye, 5.567 Debye, and 4.151 Debye, respectively, and the lowest ΔE of 1.272 eV, 1.070 eV, and 1.148 eV, respectively, recalling that higher TDM and lower ΔE are generally associated with greater reactivity. In contrast, Fig. 5-7–15 show yellow surfaces, indicating a neutral potential (zero charge) for various other interaction possibilities between Li and graphene. These structures likely exhibit lower reactivity compared to those presented in Fig. 5-4–6.

### Vibrational spectra of graphene/Li

To confirm the stability of the optimized molecular structures, the researchers employed vibrational spectroscopy calculations. Figure 6 presents the IR spectra obtained using the DFT: B3LYP/6-31g(d,p) method. The figure compares the IR spectrum of pristine graphene to the spectra of graphene-Li composites. One important aspect to note is the absence of negative frequencies in any of the spectra. This is a positive indicator that the studied structures are optimized and stable at the chosen theoretical level (DFT: B3LYP/6-31g(d,p)). Since there is no comparison to experimental data, scaling the calculated IR spectra was not necessary. The specific assignments of the observed peaks in the spectra are provided in Table 2.

**Table 2:** IR modes with their corresponding assignments for graphene as well as graphene interacted with Li which calculated at DFT:B3LYP/6-31g(d,p).

No.	Modes	G_0	G_1	G_2	G_3	G_4	G_5	G_6	G_7	G_8	G_9	G_10	G_11	G_12	G_13
1	$\gamma C-C$	568	574	540	568	552	546	572	528	584	568	564	563	568	521
2	$\gamma C-H$	877	867	866	853	852	845	860	920	866	854	877	860	877	951
3	$\beta C-H$	1163	1193	1179	1112	1166	1154	1172	1251	1184	1115	1167	1169	1167	1164
4	$\upsilon C=C$	1666	1652	1647	1650	1548	1630	1640	1562	1666	1652	1667	1667	1667	1656
5	$\upsilon C-H$	3195	3190	3195	3204	3195	3195	3200	3208	3190	3206	3176	3201	3194	3207

Based on the combined analysis of the IR spectra in Fig. 6 and the band assignments listed in Table 2, a key vibrational mode for pristine graphene can be identified. This mode corresponds to the stretching motion of carbon–carbon bonds, appearing in the range of 3300–3100 cm^−1^. Analysis of the vibrational spectra (likely referring to Fig. 6 and Table 2) suggests that Mode 5 corresponds to a stretching motion within the molecule, detectable in the wavenumber range of 1800–1600 cm^−1^. The specific peak for this mode is positioned at 3196 cm^-1^. The introduction of Li to graphene influences its vibrational fingerprint, as revealed by spectral analysis (likely Table 2). This influence can be categorized into three main effects on different vibrational modes: In-plane bending: Li causes a redshift (lower wavenumber) for some graphene configurations (G_1, G_6, G_8) in this mode, while others experience a blue shift (higher wavenumber). Out-of-plane bending: Only configurations G_7 and G_13 exhibit a redshift upon Li addition, with the rest remaining blue shifted. Stretching: Interestingly, Li induces redshifts in the stretching vibrations for pristine graphene in specific configurations (G_10–G12). Conversely, most other configurations experience blue shifts. Li also affects the wavenumbers of certain pristine graphene modes. In some configurations (G_3, G_6, G_7, G_9, G_11, G_13), Li allows these modes to vibrate at higher wavenumbers. However, the opposite happens for the remaining configurations, where the modes are shifted to lower wavenumbers.

### Physical parameters of graphene/Li/sodium alginate

The research explores the possibility of further functionalizing graphene models previously studied (G/Li and G/2Li) with SA. Figure 7 presents the model molecules for this investigation; (a) SA alone, (b) Graphene functionalized with SA (graphene/SA), (c) Graphene/SA combined with lithium weakly interacting with two carbons (graphene/SA/Li), (d) Graphene/SA combined with Li weakly interacting with two side carbons (graphene/SA/Li). These structures were optimized using a computational method called DFT: B3LYP/6-31g(d,p). The next step involved calculating key properties like TDM and ΔE for these models. The calculated values will likely be presented in Table 3.

**Table 3:** Total dipole moment TDM as Debye, HOMO/LUMO band gap energy ΔE as eV for the studied Sodium alginate (SA), Graphene/SA, Graphene/SA/Li weakly interacted with two carbons and Graphene/SA/Li weakly interacted with two side carbons which calculated at DFT:B3LYP/6-31g(d,p).

Structure	TDM (Debye)	ΔE (eV)
Sodium alginate (SA)	3.627	1.647
Graphene/SA	5.572	1.986
Graphene/2SA	4.002	0.252
Graphene/SA/Li weakly interacted with two inner carbons	3.947	1.392
Graphene/SA/Li weakly interacted with two side carbons	4.019	0.538
Graphene/3SA/Li weakly interacted with two side carbons	15.509	0.280

**Figure 8:**
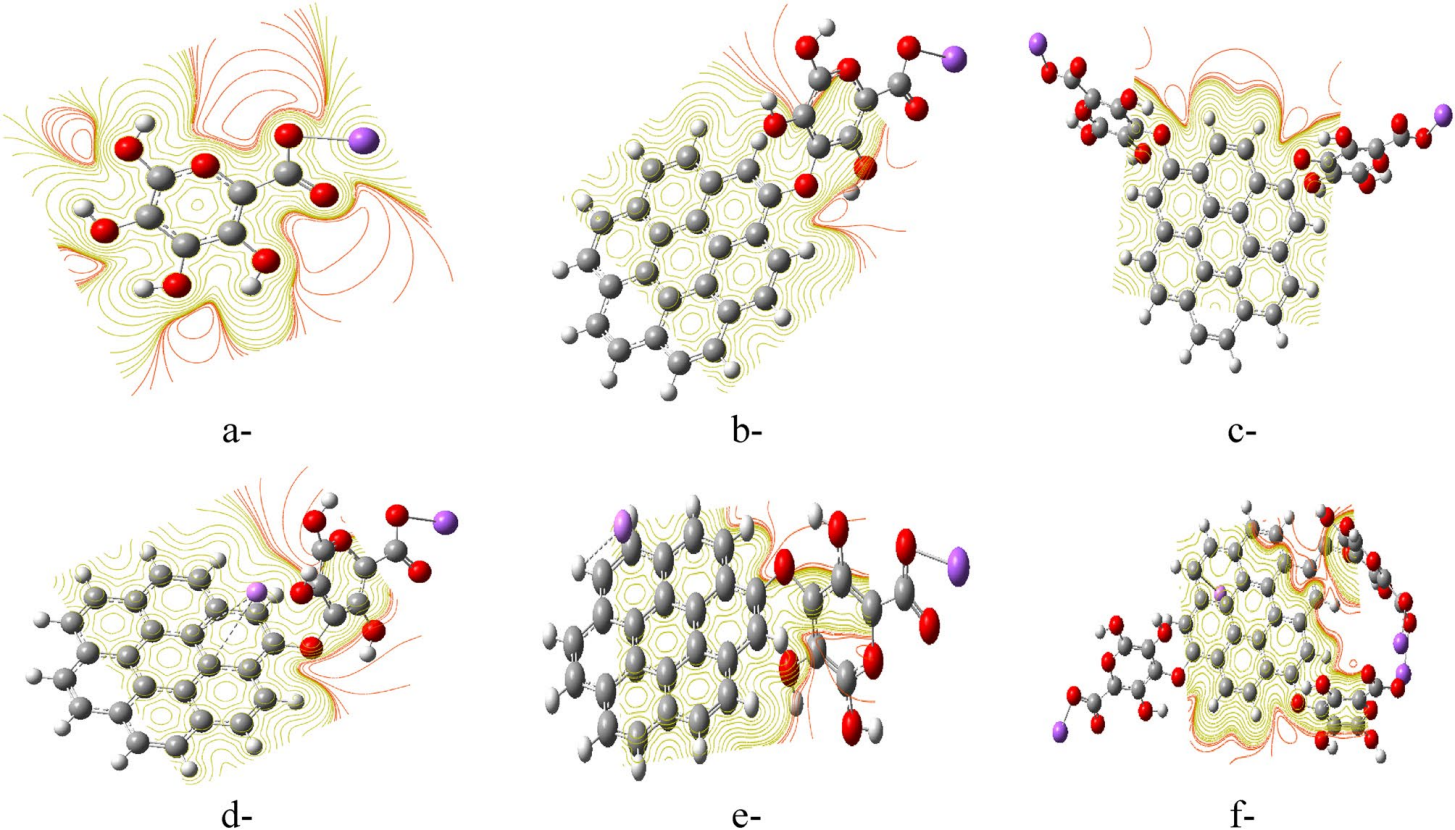
Mapping molecular electrostatic potential MESP for (**a**) SA, (**b**) Graphene/ SA, (**c**) Graphene/2SA, (**d**) Graphene/SA/Li weakly interacted with two inner carbons, (**e**) Graphene/SA/Li weakly interacted with two side carbons and (**f**) Graphene/SA/Li weakly interacted with two side carbons. All structures are calculated at DFT:B3LYP/6-31g(d,p) level of theory.

Analysis of the calculated properties (likely from Table 3) reveals the most reactive structure to be graphene functionalized with three SA units and lithium weakly interacting with two side carbons (Fig. 7d). This structure exhibits a significantly increased TDM of 15.509 Debye compared to the other models. Additionally, ΔE is significantly reduced to 0.280 eV, indicating greater reactivity. Figure 8 depicts the MESP maps for these structures. The maps suggest that SA increases the potential density of graphene, particularly in areas close to the alginate molecules. This effect is most pronounced in the structure of three SA units, aligning well with its exceptional reactivity observed in Table 3.

Table 4 presents the calculated vibrational frequencies (IR modes) and their assignments for various models using a computational method (DFT: B3LYP/6-31g(d,p)). These models include pristine graphene (G) and graphene interacting with different combinations of SA and lithium (Li); G/NaAlg: SA attached to graphene, G/2NaAlg: Graphene with two SA units, G/NaAlg/Li inner: SA, graphene, and Li (Li weakly interacting with two inner carbons of graphene), G/NaAlg/Li side: SA, graphene, and Li (Li weakly interacting with two side carbons of graphene), G/3NaAlg/Li side: Graphene with three SA units and Li (Li weakly interacting with two side carbons). The analysis of these vibrational modes suggests that introducing SA to graphene (except for the case with three SA units and side-interacting Li) does not follow a clear trend in terms of wavenumber shifts. However, graphene itself seems to be affected by the interaction with SA. In contrast, the model with three SA units and side-interacting Li (G/3NaAlg/Li side) exhibits distinct behavior. Compared to pristine graphene, the four characteristic bands in its vibrational spectrum experience a shift towards lower wavenumbers (redshift).

**Table 4:** IR modes with their corresponding assignments for graphene as well as graphene interacted with Li/SA.

No.	G	G/NaAlg	G/2NaAlg	G/NaAlg/Li_inner_	G/NaAlg/Li_side_	G/3NaAlg/Li_side_	Modes
1	568	591	559	547	554	546	$\gamma C-C$
2	877	871	891	879	882	869	$\gamma C-H$
3	1163	1167	1192	1168	1170	1151	$\beta C-H$
4	1666	1664	1667	1656	1651	1634	$\upsilon C=C$
5	3195	3193	3193	3195	3193	3177	$\upsilon C-H$

G: Graphene, G/SA: Graphene/SA, G/2SA:Graphene/2SA, G/SA/Li_inner_: Graphene/SA/Li weakly interacted with two inner carbons, G/SA/Li_side_: Graphene/SA/Li weakly interacted with two side carbons and G/SA/Li_side_: Graphene/3SA/Li weakly interacted with two side carbons. All structures are calculated at DFT:B3LYP/6-31g(d,p) level of theory.

### Global reactivity descriptors for the studied structures

This study investigated how easily molecules can lose or gain electrons (ionization potentials and electron affinities) using various methods and a specific set of conditions (6–31 + G** basis set). The results are summarized in Table 5. In general, molecules with high ionization energies are stable and less reactive, while molecules with low ionization energies are more reactive^51^. This part examined how adding Li atoms affected the ionization energy (IP) of graphene/SA. Surprisingly, even though Li only weakly interacts with two carbon atoms on the edge of graphene, it caused the IP to reach its highest measured value (6.871 eV). This unexpected result suggests that Li-modified graphene might be more reactive than the other components studied^52^. The electron affinity of graphene/SA when it interacts with Li atoms by various methods was also investigated. Interestingly, the calculated results reported that graphene has the highest electron affinity when Li weakly interacts with two carbon atoms on its edge. The study also calculated the electronic chemical potential (μ) of the molecules. They categorized the molecules based on their energy gap. Molecules with a large energy gap are considered “hard” and more difficult to polarize. This is because exciting electrons in these hard molecules requires a lot of energy. Conversely, molecules with a small ΔE are called “soft” and are more easily polarized^53^. Utilizing Koopman’s theorem for closed-shell compounds, the electronic chemical potential (μ), chemical hardness (η), and absolute softness (S) can be formally defined, $\mu =\frac{-(I+A)}{2}$, $\eta =\frac{1-A}{2}$ , and $S=\frac{1}{2\eta }$, where I and A are the ionization potential and electron affinity of the compounds, respectively.

**Table 5:** Global reactivity descriptors for the studied structure which calculated at DFT:B3LYP/6-31g(d,p).

Structure	Ionization Potential (I)	Electronic Affinity (A)	Electronic chemical potential (μ)	Chemical hardness (η)	Absolute softness (S)	Electrophilicity index (ω)
Sodium alginate (SA)	2.933	1.283	− 2.108	0.825	1.212	2.692
Graphene/SA	3.385	1.400	− 2.393	0.993	1.007	2.883
Graphene/2SA	2.385	2.133	− 2.259	0.126	7.929	20.234
Graphene/SA/Li weakly interacted with two inner carbons	3.052	1.660	− 2.356	0.696	1.437	3.990
Graphene/SA/Li weakly interacted with two side carbons	6.871	6.271	− 6.571	0.3	3.333	71.963
Graphene/3SA/Li weakly interacted with two side carbons	3.727	2.374	− 3.05	0.677	1.478	6.878

This study used two measures, absolute hardness and softness, to assess how adding Li atoms affects the stability and reactivity of graphene/SA. They found that the hardness and chemical potential of the composite changed depending on where the Li atoms were attached. This change indicates that the interaction with Li makes the graphene/SA more reactive. Parr et al.^54^ developed a new way to measure a molecule’s overall ability to accept electrons, called the electrophilicity index (ω). This index reflects how much energy is released when the molecule gains electrons through the most efficient possible electron transfer from a donor molecule. The definition of the electrophilicity index (ω) by Parr et al. is $=\frac{{\mu }^{2}}{2\eta }$. The computed electrophilicity index value characterizes the biological activity of graphene/SA when interacting with Li atoms at different positions. Interestingly, Table 5 shows that when Li atoms interact with the graphene/SA composite, the electrophilicity index increases. This effect is especially pronounced when Li weakly interacts with just two carbon atoms on the edge of the graphene sheet (with a value of 71.963). This increase in electrophilicity suggests that the interaction with Li makes the composite more likely to accept electrons. Modifying SA with graphene and Li atoms could significantly improve its usefulness in medicine by making it more functional, compatible with living tissues, and adaptable for a wider range of applications^55–57^.

### Density of states

**Figure 9:**
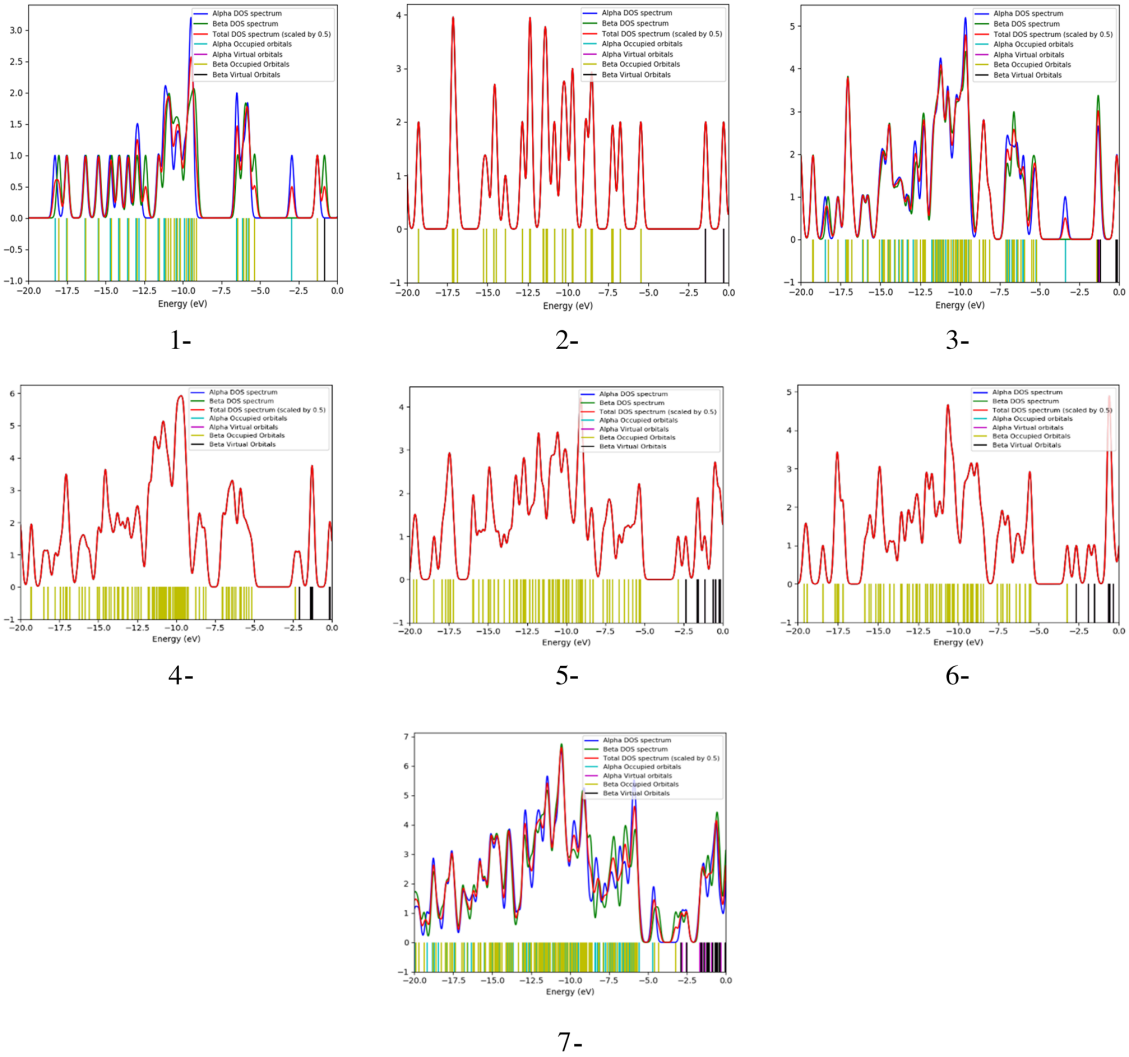
DOS for the studied structures for 1- Sodium alginate (SA), 2- Graphene, 3- Graphene/SA, 4- Graphene/2SA, 5- Graphene/SA/Li weakly interacted with two inner carbons, 6- Graphene/SA/Li weakly interacted with two side carbons and 7- Graphene/3SA/Li weakly interacted with two side carbons. All structures are calculated at DFT:B3LYP/6-31g(d,p) level of theory.

**Figure 10:**
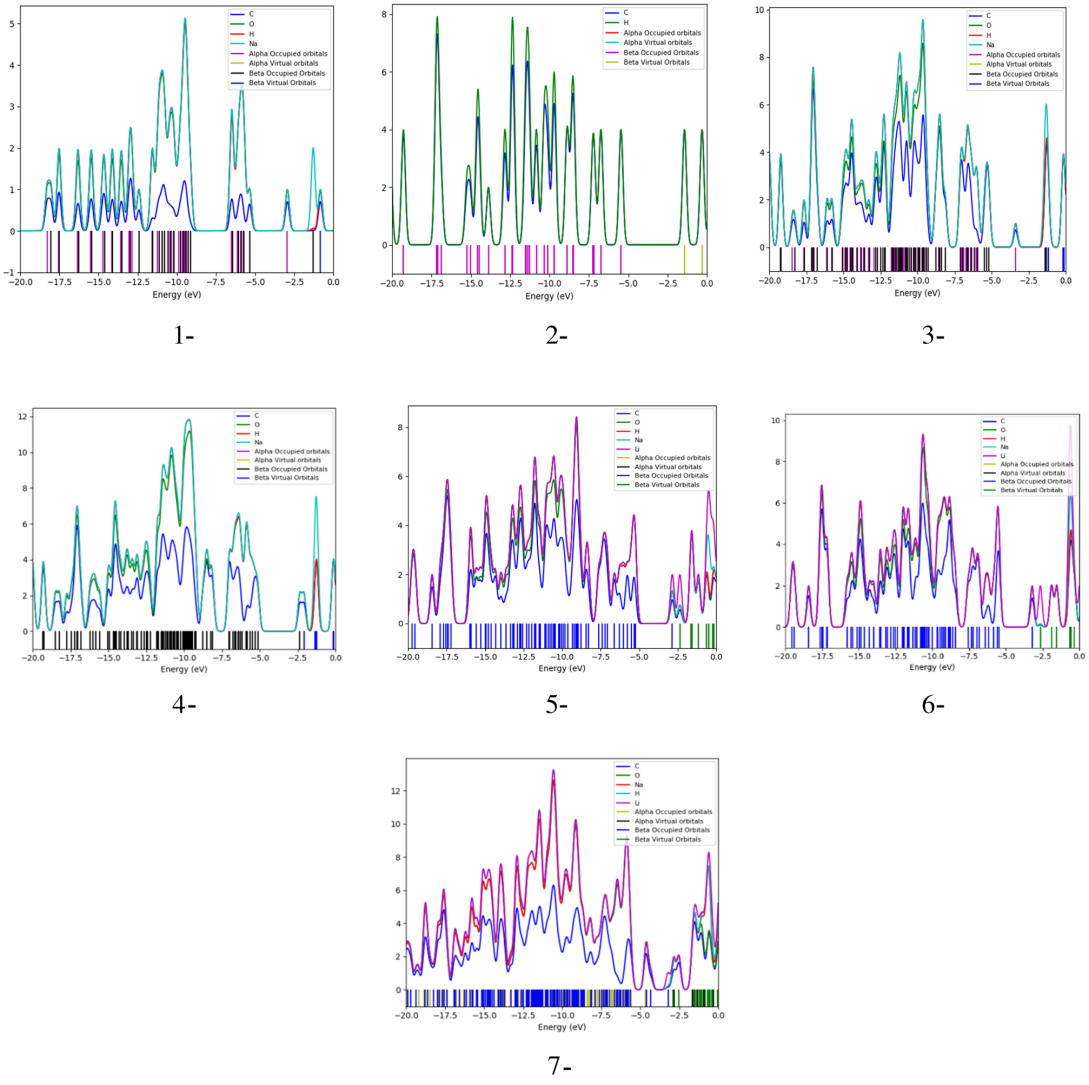
PDOS for the studied structures for 1- Sodium alginate (SA), 2- Graphene, 3- Graphene/SA, 4- Graphene/2SA, 5- Graphene/SA/Li weakly interacted with two inner carbons, 6- Graphene/SA/Li weakly interacted with two side carbons and 7- Graphene/3SA/Li weakly interacted with two side carbons. All structures are calculated at DFT:B3LYP/6-31g(d,p) level of theory.

In order to have better insight into the modification of the electronic properties of graphene following functionalization with sodium alginate (SA), Figs. 9 and 10 present the density of states (DOS) and projected density of states (PDOS), respectively, for the studied structures. DOS and PDOS can be simply identified as projecting the wavefunctions onto the atomic orbitals, and then one can compute the density of states using only certain components. PDOS is important to understand the electronic properties of graphene as a result of functionalization with SA.

As seen in Fig. 10-1, which shows the PDOS plot of SA, sodium had the highest contribution in the molecular orbitals of the SA molecule, while the lowest contribution in the molecular orbitals was from carbon atoms. In the PDOS plot of graphene shown in Fig. 10-2 both hydrogen and carbon had almost the same contribution in both HOMO and LUMO molecular orbitals. The PDOS plot of graphene/SA demonstrated in Fig. 10-3, indicates that the highest contribution in the HOMO and LUMO molecular orbitals of the graphene/SA structure is from sodium, followed by equal contributions from carbon, hydrogen, and oxygen. On the other hand, the PDOS plot of graphene/2 SA shown in Fig. 10-4, indicated that the highest contribution in the HOMO orbitals was from sodium, hydrogen and oxygen, and the lowest contribution was from the carbon atoms, while in the LUMO orbitals, the highest contribution was from sodium, followed by a much lower yet equal contribution from carbon, hydrogen and oxygen.

The introduction of lithium atoms into the graphene/SA structure either through two inner or two side carbons resulted in the same behavior in the PDOS plots shown in Fig. 10-5, -6, respectively, where the highest contribution in the HOMO orbitals was offered equally by lithium, sodium, hydrogen, and oxygen, while the lowest contribution was offered by carbon. In the LUMO orbitals, the highest contribution came from lithium, while the lowest contribution was from sodium, hydrogen, carbon and oxygen. Finally, the PDOS plot shown in Fig. 10-7, of graphene/3SA/Li, the highest contribution in the HOMO orbitals was equally from lithium, sodium, hydrogen, and oxygen, while the lowest contribution was from carbon. In the LUMO orbitals, the highest contribution came from lithium, followed by a lower contribution from sodium, hydrogen, and oxygen, while the lowest contribution was carbon.

## Conclusion

In conclusion, this study has elucidated the potential of functionalized graphene, particularly through its interaction with easily ionizable elements like Li and the biopolymer SA, for biomedical applications. Employing DFT at the B3LYP/6-31G(d,p) level, we extensively explored the structural, electronic, and spectroscopic properties of these graphene-based composites. These findings reveal that graphene exhibits weak interactions with Li, showcasing heightened reactivity as evidenced by its TDM and ΔE. Electrostatic potential mapping underscored the augmented potential density on the graphene surface upon enhancement with Li and SA, supported by other investigated physical properties. Remarkably, the configuration of graphene/3SA/Li exhibited the highest reactivity, emphasizing the potential for practical biomedical applications. Additionally, spectral shifts in graphene characteristics towards lower wavenumbers were observed as a consequence of Li and SA interactions. This may be attributed to the graphene interacting with Li and alginate showing changes in its ΔE values. This finding is supported by the electronic properties indicated by both DOS and PDOS. These changes may lead to a change in the optical absorption of graphene which in turn shifts the IR bands of graphene as a result of interaction with Li and alginate.

By illustrating parameters such as TDM, ΔE, molecular electrostatic potential, DOS, PDOS, and IR spectra, this study offers a comprehensive understanding of functionalized graphene systems.

## Data Availability

The data that support the findings of this study are available from the corresponding author upon reasonable request.
